# Ultrastable Covalent Triazine Organic Framework Based on Anthracene Moiety as Platform for High-Performance Carbon Dioxide Adsorption and Supercapacitors

**DOI:** 10.3390/ijms23063174

**Published:** 2022-03-15

**Authors:** Mohamed Gamal Mohamed, Santosh U. Sharma, Ni-Yun Liu, Tharwat Hassan Mansoure, Maha Mohamed Samy, Swetha V. Chaganti, Yu-Lung Chang, Jyh-Tsung Lee, Shiao-Wei Kuo

**Affiliations:** 1 Department of Materials and Optoelectronic Science, Center for Functional Polymers and Supramolecular Materials, National Sun Yat-sen University, Kaohsiung 80424, Taiwan; mgamal.eldin12@aun.edu.eg (M.G.M.); m083100007@nsysu.edu.tw (N.-Y.L.); d083100006@nsysu.edu.tw (M.M.S.); 2 Department of Chemistry, Faculty of Science, Assiut University, Assiut 71516, Egypt; tharout.mansour@science.au.edu.eg; 3 Department of Chemistry, National Sun Yat-sen University, Kaohsiung 80424, Taiwan; sksharma25086@g-mail.nsysu.edu.tw (S.U.S.); d082630006@g-mail.nsysu.edu.tw (S.V.C.); yulung@g-mail.nsysu.edu.tw (Y.-L.C.); 4 Department of Medicinal and Applied Chemistry, Kaohsiung Medical University, Kaohsiung 80708, Taiwan

**Keywords:** anthracene, covalent triazine frameworks (CTFs), CO_2_ uptake, supercapacitors

## Abstract

Conductive and porous nitrogen-rich materials have great potential as supercapacitor electrode materials. The exceptional efficiency of such compounds, however, is dependent on their larger surface area and the level of nitrogen doping. To address these issues, we synthesized a porous covalent triazine framework (An-CTFs) based on 9,10-dicyanoanthracene (An-CN) units through an ionothermal reaction in the presence of different molar ratios of molten zinc chloride (ZnCl_2_) at 400 and 500 °C, yielding An-CTF-10-400, An-CTF-20-400, An-CTF-10-500, and An-CTF-20-500 microporous materials. According to N_2_ adsorption–desorption analyses (BET), these An-CTFs produced exceptionally high specific surface areas ranging from 406–751 m^2^·g^−1^. Furthermore, An-CTF-10-500 had a capacitance of 589 F·g^−1^, remarkable cycle stability up to 5000 cycles, up to 95% capacity retention, and strong CO_2_ adsorption capacity up to 5.65 mmol·g^−1^ at 273 K. As a result, our An-CTFs are a good alternative for both electrochemical energy storage and CO_2_ uptake.

## 1. Introduction

Over the last few decades, there has been a dramatic surge in the need for future sustainable energy. The cause for this massive demand is the variety of natural resources that supply raw materials to support industrialization and urbanization in our modern era [1,2,3,4,5]. This trend has raised major concerns about the availability of natural resources for future generations, which might lead to disaster in terms of global warming and a shortage of energy supplies [5,6,7,8,9,10,11,12]. To address these issues, researchers across the world have devoted themselves to finding clean and sustainable electrical energy storage devices. Thus, one of the most important energy storage devices discovered is the electric double-layer capacitor (EDLC), also known as a supercapacitor, which piqued the interest of many people due to its substantial power density, high energy density, a wide range of operating temperature, and consistent cycling performance over time [12,13,14,15]. The key point of the working mechanism of these supercapacitors is that they store the charged particles between the interface of electrode and electrolyte with the help of dynamic electrolyte ions. Thus, the performance of these supercapacitors depends on the accessible area of the electrode material with a high surface area and porous morphology to increase the movement of electrolyte ions [12,13,14,15,16,17]. However, the low energy density (about 10 W·h·kg^−1^) of carbon-based supercapacitors remains an issue [15]. Furthermore, the rate performance at high current densities is poor, restricting their broad use in real-world scenarios for future energy storage [16,18]. As a result, one of the key research issues in the field is the development of supercapacitor devices with high rate and energy density. The energy density of carbon-based supercapacitors is directly connected to the specific capacitance of carbon nanomaterials and the voltage window [12,13,14,15,16,17,18]. As a result, increased porosity with a large surface area and strong electrical conductivity are essential components of high-performance pseudocapacitive energy storage devices. Various studies have been conducted to date in order to provide highly sustainable and efficient energy storage systems, which include metal oxides [19,20], metal–organic framework (MOFs) [21,22], activated carbon [23,24,25], porous carbon and graphene [26,27,28,29], porous organic polymer [30,31,32,33,34], and other carbon-based materials [35,36]. Because of their higher mechanical stability, low densities, higher surface area, and greater extent of porosity, newly developed porous organic polymers and covalent triazine frameworks (CTFs) have been widely discussed, making this type of material very unique in providing highly efficient energy storage devices and other potential applications [37,38,39,40,41,42,43]. CTF precursors are materials containing nitrogen, which are easily formed by cyclotrimerization of aryl nitriles under ionothermal conditions [44]. CTFs have been used in heterogeneous catalysis, gas storage, CO_2_ absorption and conversion, and sensing, as well as electrocatalysis for oxygen reduction reactions, potential electrodes in lithium batteries, supercapacitors, and other energy storage systems [45,46,47,48,49,50]. Vadiyar et al. reported a polyethynylbenzonitrile-based CTFs with high specific surface area, higher capacity retention (71%), and high capacitance up to 628 F·g^−1^ at 50 A·g^−1^ [51]. Li et al. successfully synthesized nitrogen-enriched tetracyanoquinodimethane-derived conductive CTFs (TCNQ-CTFs), which also exhibited very good specific capacity (380 F·g^−1^), high energy density (42.8 W·h·kg^−1^), and extraordinary cycling performance up to 10,000 cycles without compromising the loss of capacity [52]. In addition to this, Bhanja et al. prepared a covalent organic framework (COF) based on triazine units. The obtained polymer exhibited a specific capacitance of 354 F·g^−1^ because of high conjugation and large porosity. The compound also delivered very high cycling stability with 95% retention of the capacity after several thousand cycles [53].

**Figure 1:**
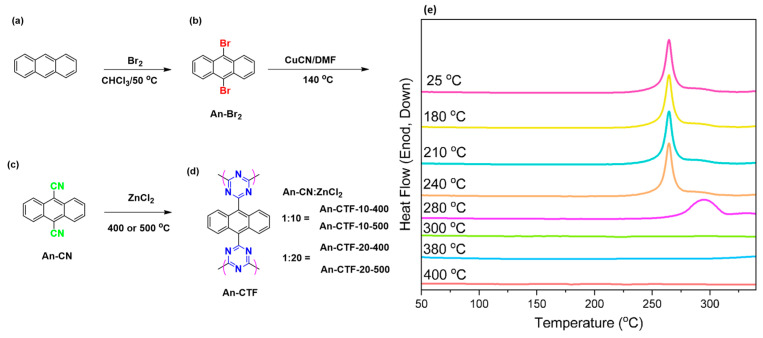
Preparation way and reaction conditions of An-Br_2_ (**b**), An-CN (**c**), and An-CTFs (**d**) from An (**a**). (**e**) DSC profile of An-CN monomer at various thermal treatments from 25 to 400 °C.

Therefore, we synthesized a new class of nitrogen-enriched covalent organic framework based on An units in the presence of ZnCl_2_ at two different temperatures (400 °C and 500 °C) using two different molar ratios (0.1 and 0.05) of An-CN/ZnCl_2_ (Figure 1). We characterized these AN-CTFs using Fourier-transform infrared spectroscopy (FTIR), thermogravimetric analysis (TGA), wide-angle X-ray diffraction (WAXD), Raman spectroscopy, X-ray photoelectron spectroscopy (XPS), scanning electron microscopy (SEM), transmission electron microscopy (TEM), and Brunauer–Emmett–Teller (BET) theory to investigate the different properties of such frameworks such as chemical compositions, porosity, thermal stability, crystallinity, surface area, and chemical structure. In addition, we also evaluated the electrochemical performance for energy storage, as well as CO_2_ uptake for gas storage applications. 

## 2. Results and Discussion

### 2.1. Synthesis and Characterization of An-CN and An-CTFs

As known, the anthracene (An) unit consists of three fused benzene rings with highly planar conjugation structure, and it is widely used in field-effect transistors, solar cells, and light-emitting diodes (LED). The An-CTFs were synthesized in three steps as shown in Figure 1. Firstly, anthracene (Figure 1a) was converted into 9,10-dibromoanthracene (An-Br_2_) using Br_2_ in chloroform (Figure 1b) at 50 °C for 4 h. Then, An-Br_2_ was reacted with CuCN in dry DMF to give 9,10-cyanoanthracene (An-CN) (Figure 1c). Then we used the ionothermal method for trimerization of CN groups of An-CN in anhydrous molten ZnCl_2_ with different CN-An/ZnCl_2_ ratios at two different reaction temperatures of 400 and 500 °C, yielding An-CTF-10-400, An-CTF-20-400, An-CTF-10-500, and An-CTF-20-500 (Figure 1d). The presence of nitrile groups and their cyclotrimerization reactions in An-CN were confirmed through DSC analysis (Figure 1e). The DSC profile of the An-CN monomer before and after thermal treatment from 25 to 240 °C displayed a maximum exothermic peak of the CN group at 265 °C. We found that the maximum exothermic peak of CN groups was shifted to 295 °C and completely absent when the temperature reaction was 300, 340, 380, and 400 °C, due to the formation of triazine rings and completion of the cyclotrimerization reaction to form An-CTFs precursors. 

**Figure 2:**
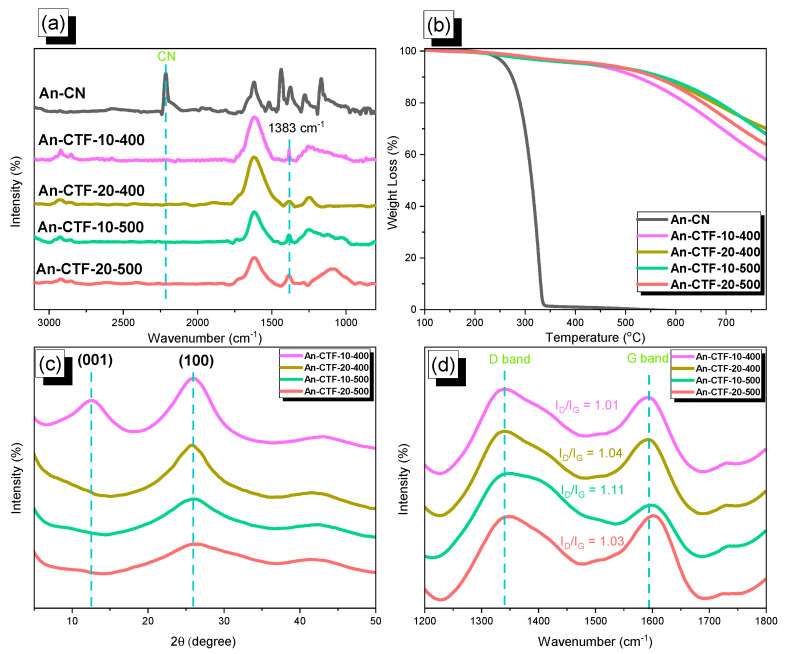
FTIR (**a**), TGA (**b**), XRD (**c**), and Raman (**d**) analyses of An-CN, An-CTF-10-400, An-CTF-20-400, An-CTF-10-500, and An-CTF-20-500.

The FTIR spectra of An-CN and its corresponding synthesized CTFs at different temperatures are displayed in Figure 2a. The FTIR profile of An-CN featured a band at 2220 cm^−^^1^ corresponding to the CN unit, which also confirmed its synthesis. Furthermore, as shown in Figure 2a, the FTIR spectra of An-CTFs exhibited absorption bands at 1566 and 1383 cm^−^^1^ attributed to the triazine rings in each CTF without any bands of CN, confirming the trimerization or cyclization of the nitrile groups [54,55]. To gain insight into the thermal stability of as-prepared CTFs and An-CN, we performed TGA analysis under N_2_ atmosphere from 40–800 °C. As seen in Figure 2b and Table 1, the decomposition temperature (T_d10_) of An-CTF-10-500 was 573 °C, which was the highest among other An-CTFs with a higher char yield of 66 wt.%. The decomposition temperatures of other An-CTFs were smaller compared to An-CTF-10-500 with char yields of 56, 69, and 62 wt.% for An-CTF-10-400, An-CTF-20-400, and An-CTF-20-500, respectively. Therefore, An-CTF-10-500 had a superior decomposition temperature and high char yield, making it more thermally stable. On the other hand, the degrading temperature of the An-CN monomer was 274 °C with no char yield. Overall, our new An-CTFs had excellent thermal stability. Figure 2c represents the XRD patterns of An-CTFs in the range of 5–50 °C. Upon seeing the spectrum, the two broad peaks located at 12 and 26 °C could be attributed to a partially crystalline structure, assigned to the (001) and (100) plane indices [56,57,58,59]. Raman spectroscopy is considered an important method to examine and investigate the graphitization degree and defect properties of carbon materials. The Raman spectra of as-prepared CTFs are shown in Figure 2d, recorded from 1200 to 1700 cm^−^^1^. The spectra of all An-CTFs showed two strong signals characteristic of the D and G bands, revealing the presence of graphitic carbonized structure. These D and G bands represent the carbonaceous materials formed from chemical structures of CTFs, as well as other carbonaceous materials used in electrode preparation. In general, they represent two kinds of hybridization (*sp*^3^ and *sp*^2^), corresponding to the second- and first-order Raman scattering. The D and G bands for all four An-CTFs were located at 1350 and 1602 cm^−^^1^, respectively [54,55]. The I_D_/I_G_ ratios for An-CTF-10-400, An-CTF-20-400, An-CTF-10-500, and An-CTF-20-500 were 1.01, 1.04, 1.11, and 1.03 respectively, suggesting that An-CTF-10-400, An-CTF-20-400, and An-CTF-20-500 had a higher degree of graphitization, indirectly revealing the formation of fewer defects in their morphology with an increase in the condensed aromatic structure when compared to An-CTF-10-500 and other reported CTFs [54,55,56,57,58,59]. An-CTF-10-500 showed a slightly lower degree of graphitization when compared with other samples, possibly due to the low amount of ZnCl_2_ and high temperature (500 °C) leading to more defects in the graphene structure.

**Figure 3:**
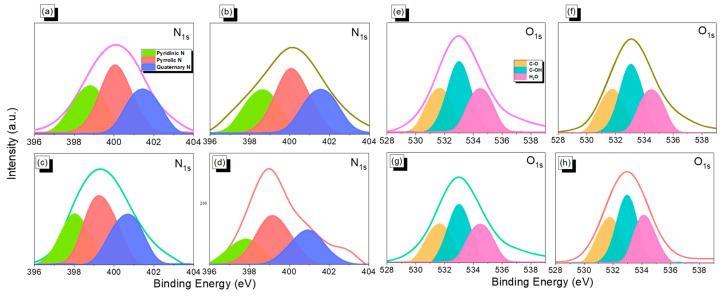
XPS fitting curves of N_1s_ (**a**–**d**) and O_1s_ (**e**–**h**) orbitals for An-CTF-10-400, An-CTF-20-400, An-CTF-10-500, and An-CTF-20-500.

The XPS spectra of all synthesized An-CTFs in this study (Figure S5) showed three peaks corresponding to the characteristic peaks of the carbon atom (at 284 eV), C–N bond for N*_1s_* orbital (at 400 eV) in triazine unit, and O*_1s_* orbital with absorbed moisture and oxygen (at 530 eV) [54,55,56,57,58,59]. In addition, Figure 3a–h and Table 2 show the fitted XPS curves for N*_1s_* and O*_1s_* orbitals to examine the chemical composition on the surface of these as-prepared An-CTFs. All of the fitted results showed the existence of three different N species: quaternary N species (401.5 eV), pyrrolic species (400 eV), and hexagonal pyridinic N atom due to the triazine unit (398.5 eV). Following a quantitative investigation, pyrrolic N was shown to be the most prevalent, whereas the remaining species of quaternary and pyridinic N were essentially identical in the synthesized CTFs. In addition to this, the three O_1s_ orbital peaks in Figure 3 reveal that the surface of these CTFs also contained three different O species such as C–O, moisture, and absorbed oxygen at 531.5, 533, and 535 eV, respectively [54,60,61]. 

### 2.2. Porosity and CO_2_ Uptake of An-CTFs

**Figure 4:**
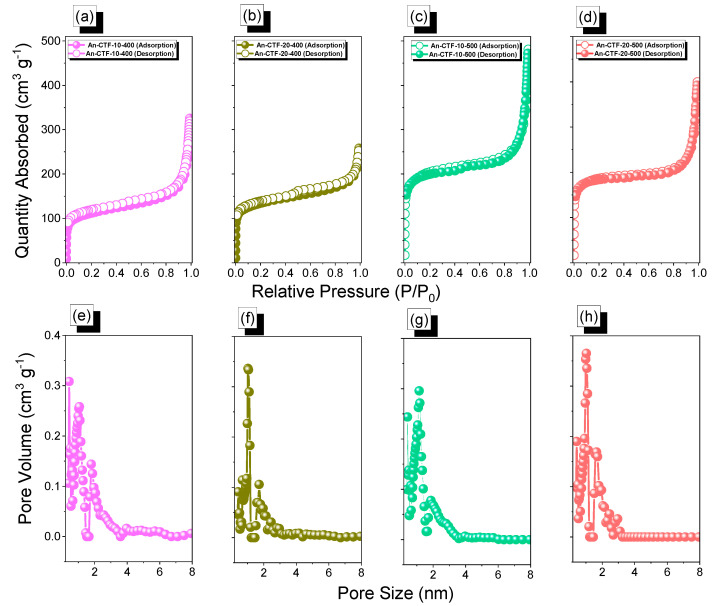
BET and pore diameters curves of An-CTF-10-400 (**a**,**e**), An-CTF-20-400 (**b**,**f**), An-CTF-10-500 (**c**,**g**), and An-CTF-20-500 (**d**,**h**).

Furthermore, we extended our study to examine the surface area and porosity of our An-CTFs materials. Figure 4 represents the BET isotherms and pore size analyses of An-CTFs at 77 K. Figure 4a–d depict the BET curves of all An-CTFs, which all had a type IV isotherm with high BET surface areas of 406, 491, 751, and 700 m^2^·g^−^^1^ for An-CTF-10-400, An-CTF-20-400, An-CTF-10-500, and An-CTF-20-500, respectively. The order of decreasing surface area was An-CTF-10-500 > An-CTF-20-500 > An-CTF-20-400 > An-CTF-10-400. Thus, the high specific surface area of An-CTF-10-500 was attributed to the availability of closely packed nanoparticles with great defects for ion conduction [54,60]. The pore diameters of all An-CTFs according to NL-DFT theory (Figure 4e–h and Table 1) were 1.01–1.84, 1.02–1.72, 1.18–1.87, and 1.06–1.66 nm for An-CTF-10-400, An-CTF-20-400, An-CTF-10-500, and An-CTF-20-500, respectively.

**Figure 5:**
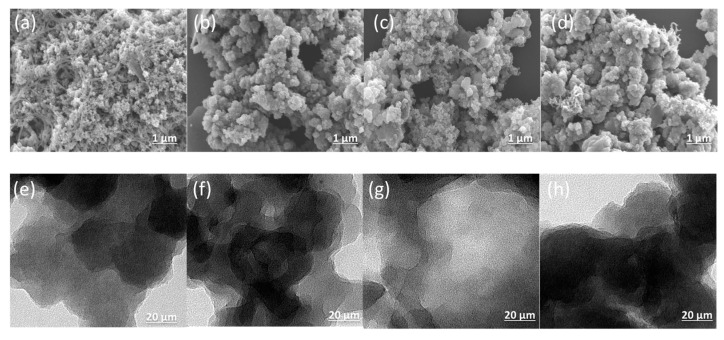
SEM and TEM images of An-CTF-10-400 (**a**,**e**), An-CTF-20-400 (**b**,**f**), An-CTF-10-500 (**c**,**g**), and An-CTF-20-500 (**d**,**h**).

Figure 5a–d show the SEM images taken for the An-CTFs. As seen in SEM images, An-CTFs had regular and uniform spherical particles with small nanorods. Furthermore, HR-TEM analysis (Figure 5e–h) of An-CTFs materials revealed the presence of irregular micropores. The SEM-EDS mapping (Figure S6) results revealed the presence and dispersion of C, N, and O atoms in An-CTF-10-400 and An-CTF-10-500. 

**Figure 6:**
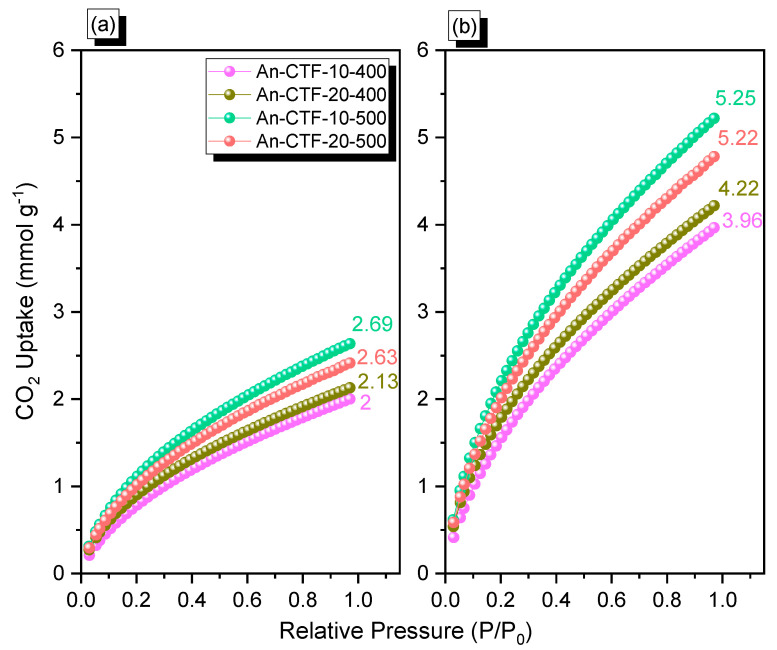
CO_2_ adsorption performance of An-CTF-10-400, An-CTF-20-400, An-CTF-10-500, and An-CTF-20-500, recorded at 298 K (**a**) and 273 K (**b**).

To investigate the potential of An-CTFs for CO_2_ capture, we performed CO_2_ adsorption isotherms for all CTFs at 298 and 273 K. The as-obtained CO_2_ isotherms are presented in Figure 6 and Table 1. The CO_2_ capture results of all four An-CTFs at 298 K (Figure 6a) were 2.00, 2.13, 2.63, and 2.69 mmol·g^−^^1^ for An-CTF-10-400, An-CTF-20-400, An-CTF-10-500, and An-CTF-20-500, respectively. At 273 K (Figure 6b), the CO_2_ capacity of An-CTF-10-400, An-CTF-20-400, An-CTF-10-500, and An-CTF-20-500 was found to be 3.96, 4.22, 5.22, and 5.25 mmol·g^−^^1^, respectively. Thus, An-CTF-10-500 demonstrated a higher degree of CO_2_ uptake compared to other CTFs. An-CTF-10-500 was superior not only to other An-CTFs in this study but also to other reported materials. An-CTF-10-500 showed higher CO_2_ uptake capability than other CTF precursors such as Car-CTF-10-500 [54], P-CTF1-3 [62], and CTF-0 [45], attributed to its high pore diameter and high S_BET_ of 751 m^2^·g^−^^1^, which helped to increase the binding affinity between CO_2_ and the An-CTF-10-500 framework [54,55,60]. 

### 2.3. Electrochemical Performance of An-CTFs

**Figure 7:**
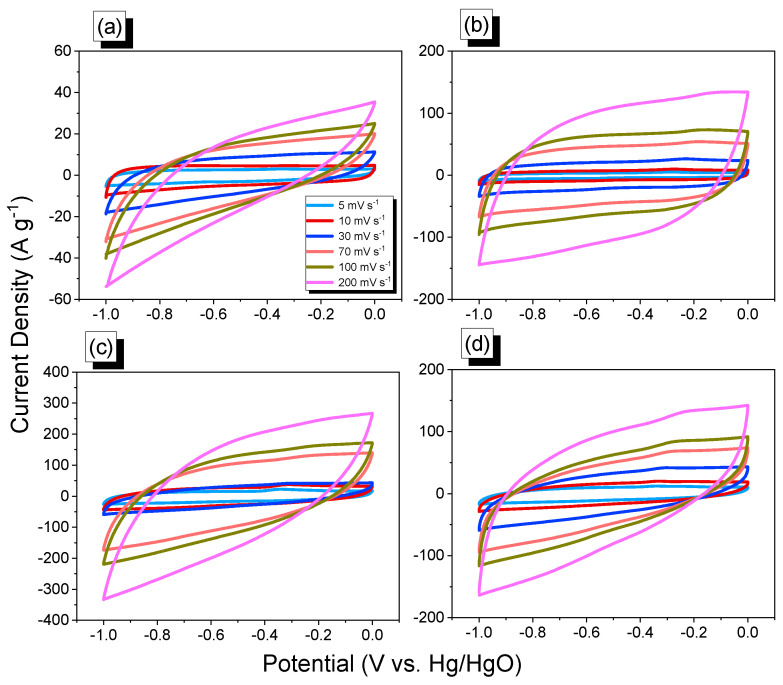
Cyclic voltammetry profiles of An-CTF-10-400 (**a**), An-CTF-20-400 (**b**), An-CTF-10-500 (**c**), and An-CTF-20-500 (**d**).

The electrochemical performance of the An-CTFs samples was determined using cyclic voltammetry (CV) and galvanostatic charge–discharge (GCD) measurements in a 1 M KOH aqueous solution utilizing a three-electrode setup. Figure 7a–d show the corresponding CV curves of the An-CTFs samples, which were recorded at various sweep speeds ranging from 5 to 200 mV·s^−^^1^ in a potential window ranging from 0 to 1.00 V (vs. Hg/HgO). The CV curves of all An-CTFs samples had rectangle-like forms, suggesting that this capacitive response was mostly caused by electric double-layer capacitance (EDLC) with little pseudo-capacitance caused by the presence of different nitrogen and oxygen species [54,55]. Furthermore, raising the molar ratio of An-CN/ZnCl_2_ from 0.05 to 0.1 at 500 °C resulted in An-CTFs materials with higher EDLC capacitance. This behavior can be explained by the reaction with ZnCl_2_, which produced a unique carbon framework with pyridinic N atoms similar to those seen in N-doped carbon materials [54,55]. BET and XPS studies demonstrated that An-CTFs samples exhibited high specific surface areas and N-heteroatom frameworks (primarily pyridinic and pyrrolic N species).

**Figure 8:**
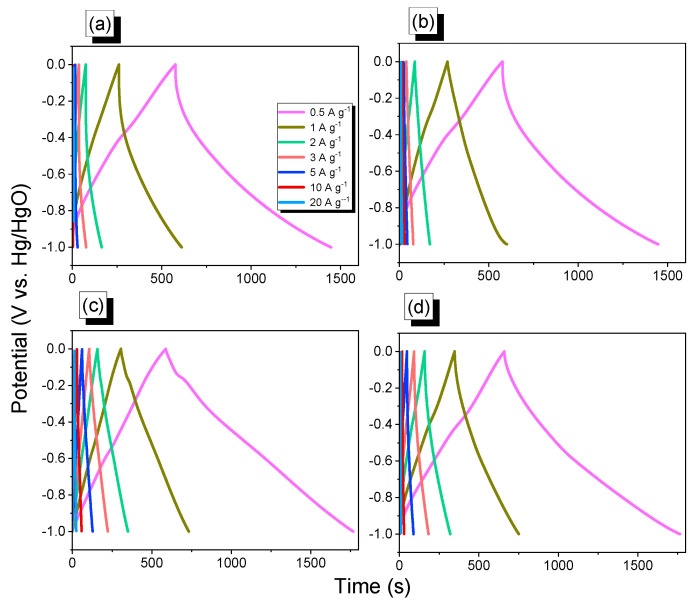
GCD profiles of An-CTF-10-400 (**a**), An-CTF-20-400 (**b**), An-CTF-10-500 (**c**), and An-CTF-20-500 (**d**).

Figure 8a–d show the GCD curves of An-CTFs samples recorded at different current densities ranging from 0.5 to 20 A·g^−^^1^. The An-CTFs sample GCD curves displayed triangular forms with a small bend, indicating both pseudo-capacity and EDLC features [54,55]. As shown in Figure 8c,d, the discharging time of An-CTF-10-500 was longer than that of the An-CTF-20-500, showing that the former’s capacitance was greater than that of the latter. 

**Figure 9:**
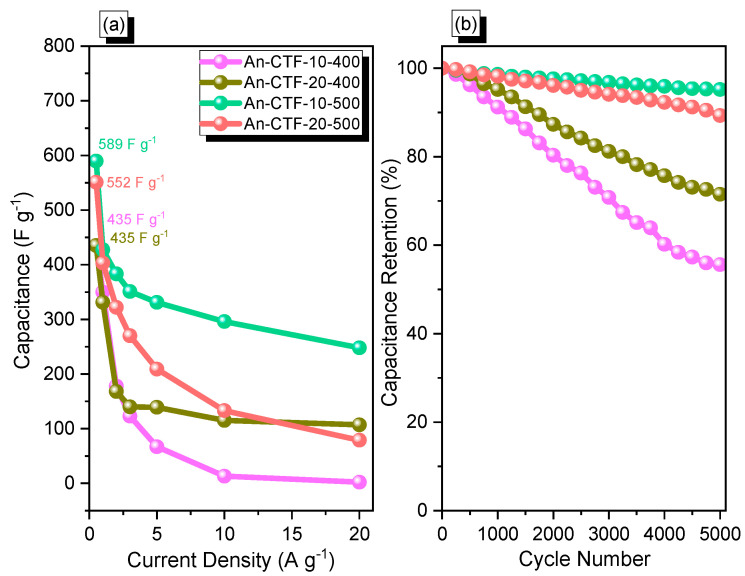
Capacitance (**a**) and cycle-life performance (**b**) of cells with An-CTF-10-400, An-CTF-20-400, An-CTF-10-500, and An-CTF-20-500.

Figure 9a and Table S1 show the specific capacitances of An-CTF samples calculated from GCD curves using Equation (S1). At a current density of 0.5 A·g^–1^, An-CTF-10-500 demonstrated good capacitance with a value of 589 F·g^–1^. The existence of graphitic microporous carbon structures with diverse functionalized units (pyridinic and pyrrolic N atoms, C=O, and phenolic OH groups) might explain An-CTF-10-500’s remarkable performance. In addition to this, the superior behavior of An-CTF-10-500 could be due to the increased molar ratio of An-CN/ZnCl_2_ from 0.05 to 0.1 at 500 °C, which helped to form a carbon-rich network with high N content. Consequently, An-CTF-20-500 also exhibited excellent specific capacitance quite similar to that of An-CTF-10-500; however, due to the low molar ratio of An-CN/ZnCl_2_, it could not outperform An-CTF-10-500. As shown in Figure 9a, the specific capacitance of all four samples, i.e.*,* An-CTF-10-400, An-CTF-20-400, An-CTF-10-500, and An-CTF-20-500, decreased upon varying the current density from 0.5 to 20 A·g^−^^1^. This unique behavior of these An-CTF materials can be attributed to the rapid adsorption of electrolyte ions on the electrodes. Furthermore, An-CTF-10-500 was superior owing to its highest surface area during the cycling performance. In addition, to examine the stability of these as-prepared electrode materials over long cycling tests, we investigated the cycling stability profiles of all An-CTF materials with GCD evaluation at a current density of 10 A·g^−^^1^ over 5000 cycles (Figure 9b). The results revealed their extraordinary stability in 1 M KOH electrolyte as An-CTF-10-400, An-CTF-20-400, An-CTF-10-500, and An-CTF-20-500 retained 56%, 72%, 95%, and 90% of their initial capacity. We previously reported two ultrastable conductive CTFs based on pyrene, and these materials exhibited high capacitance of 380 and 500 F·g^−^^1^. These high capacitances were attributed to their high specific surface areas of 819 and 1019 m^2^·g^−^^1^ and high N content [55]. Additionally, Hao et al. reported 2D microporous CTFs with suitable low capacitance (151.3 F·g^−^^1^ at 0.1 A·g^−^^1^) [57]. Thus, these An-CTFs materials show great potential to be employed as electrochemical energy storage systems. 

**Figure 10:**
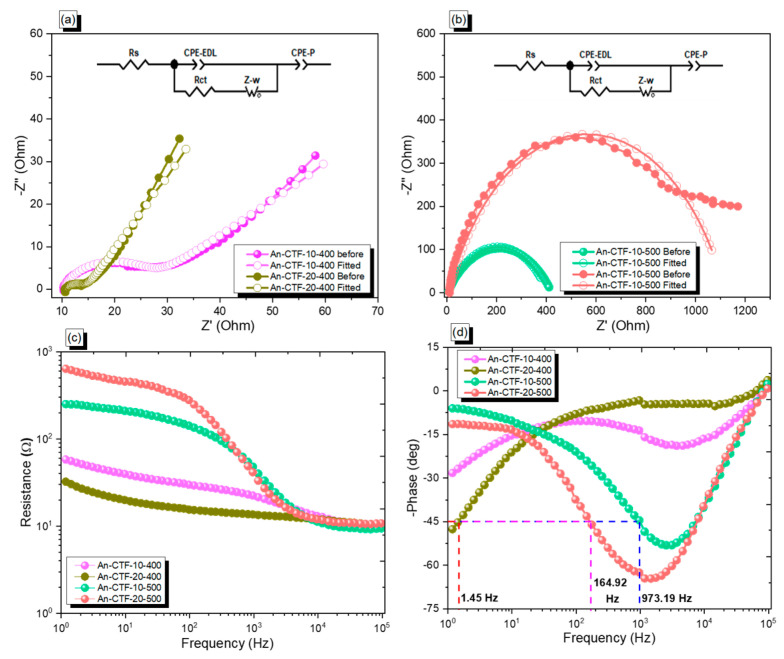
(**a**,**b**) Nyquist plots, (**c**) Bode magnitude plots, and (**d**) Bode phase plots of An-CTF-10-400, An-CTF-20-400, An-CTF-10-500, and An-CTF-20-500 cells.

Figure S7 shows that the An-CTFs displayed superior capacitance performance compared to other porous materials such as Car-CTFs [54], Py-CTFs [55], TCNQ-CTFS [52], TDFP-1 [53], and TPE-CPOP-800 [63]. Electrochemical impedance spectroscopy is an outstanding characterization method to study the interface of electrodes and electrolytes in a given frequency domain. EIS provides information about the internal resistances offered by the electrode material and used electrolyte system. Figure 10 shows the Nyquist plots of as-prepared electrode materials in 1.0 M KOH electrolyte, in which the compounds An-CTF-10-500 and An-CTF-20-500 showed depressed semicircles and a sloping line at high frequencies and low frequencies, respectively, attributed to the charge transfer during the faradic reactions at the electrode/electrolyte interface [64,65]. The EIS results demonstrated that the best electric charge transfer performance was observed with compound An-CTF-10-500, followed by An-CTF-20-500, An-CTF-10-400, and An-CTF-20-400, due to the better conductivity of An-CTF-10-500. This trend matches well with the capacitance plots of all the compounds shown in Figure 9a. In order to study the impedance properties of the electrode materials, electrochemical impedance spectroscopy (EIS) was carried out, as displayed in Figure 10a,b. The equivalent circuit was fitted with the equivalent model in order to compare the charge transfer from each electrode. Rs, Rct, CPE-EDL, CPE-P, and Zw represent the equivalent series resistance, charge transfer resistance, constant phase element representing EDLC, pseudocapacitive behavior, and Warburg element, respectively. As shown in Table S2, the initial ohmic resistance values of all electrodes were 9.951, 10.75, 4.068, and 7.08 for An-CTF-10-400, An-CTF-20-400, An-CTF-10-500, and An-CTF-20-500, respectively. Among them, An-CTF-10-500 delivered the smallest ohmic resistance; thus, it was the most conductive in nature due to the charge transfer during the faradic reactions at electrode/electrolyte interface [64,65,66,67,68]. Furthermore, Figure 10c represents the frequency-dependent Bode plots of triazine-based electrodes. The figures reveal slanted lines with a negative slope at low frequency with tiny resistance at high frequency, demonstrating the ideal capacitive behavior of the as-prepared electrodes. Figure 10d shows the frequency-dependent Bode phase plot. The knee frequency can be defined as the characteristic frequency where the phase angle reaches 45°. At this stage, both the capacitive and resistance impedance become equal in magnitude. Beyond this point at higher frequency, the supercapacitors become resistive. This also indicates the relaxation time for the device to discharge its energy with efficiency greater than 50%. The knee frequency is directly proportional to the rate capability of electrode materials.

## 3. Experimental Section

### 3.1. Materials

Anthracene (An), copper(I) cyanide (CuCN), *N*,*N*-dimethylformamide (DMF), zinc chloride (ZnCl_2_), HCl solution (37%), bromine solution (Br_2_), methanol, ethanol, dichloromethane (DCM), ethylenediamine, acetone, and tetrahydrofuran (THF) were purchased from Sigma–Aldrich (Burlington, MA, USA), Acros (Fukuoka, Japan), Alfa Aesar (Lancashire, UK) and they were used as received without further purification. 

### 3.2. Preparation of 9,10-Dibromoanthracene (An-Br_2_)

A flask containing An (2 g) in chloroform and 5 mL of Br_2_ in chloroform was heated at 50 °C for 4 h. After the reaction, the yellow solid was filtered off and washed to obtain An-Br_2_ (90%). ^1^H-NMR (500 MHz, CDCl_3_, Figure S1): 8.58 (s, 2H), 7.63 (s, 2H). ^13^C-NMR (125 MHz, CDCl_3_, Figure S2): 131.63, 129.43, 128.03, 124.16.

### 3.3. Preparation of 9,10-Cyanoanthracene (An-CN)

A mixture of An-Br_2_ (2 g) and CuCN (2.13 g) was added to a flask containing 60 mL of DMF under reflux at 140 °C for 2 days. After the reaction, the beaker was cooled to room temperature and filtered to remove excess CuCN. The DMF solution was then poured into ethylenediamine and water to afford a yellow solid. The obtained solid was crystallized in a hot solution of DMF to afford An-CN as yellow needles (85%). ^1^H-NMR (500 MHz, DMSO-*d_6_*, Figure S3): 8.46 (s, 2H), 8.05 (s, 2H). ^13^C-NMR (125 MHz, DMSO- *d_6_*, Figure S4): 132.13, 131.39, 126.36, 116.29 (CN), 111.37.

### 3.4. Preparation of An-CTFs

The An-CTFs were prepared using An-CN as the monomer precursor, mixed with anhydrous molten ZnCl_2_ at molar ratios of 0.1 and 0.05. They were subjected to two different temperatures of 400 and 500 °C and maintained for 2 days under nitrogen atmosphere to afford An-CTFs (Figure 1). The obtained solid was washed with 1 M HCl, water, THF, DCM, and methanol, before drying in an oven to afford An-CTFs.

## 4. Conclusions

We discovered a new class of nitrogen-enriched covalent organic frameworks based on triazine and An in the presence of ZnCl_2_ at two distinct temperatures and molar ratios. The structural characterization demonstrated that the synthesized An-CTFs had fewer morphological differences than previously reported CTFs. These An-CTFs had thermal stability, high BET surface area and porosity, and excellent cycle stability, which enabled us to examine the CO_2_ uptake and energy storage performance of as-prepared An-CTFs. An-CTF-10-500 outperformed the other three CTFs with a higher specific capacitance of 589 F·g^−1^ and higher capacity retention of 95% over 5000 cycles. It exhibited excellent charge transport from the electrode to electrolyte and a unique higher level of CO_2_ uptake of 5.25 mmol·g^−1^ at 273 K. From the results obtained, it is well understood that the CTFs based on the An moiety can set a new bar for applications in gas and energy storage.

## Figures and Tables

**Table 1:** TGA, porosity, and CO_2_ uptake of An-CTFs.

Material	T_d5_ (°C)	T_d10_ (°C)	Char Yield (%)	Surface Area (m^2^ g^−1^)	Pore Size (nm)	CO_2_ Uptake (298 K)	CO_2_ Uptake (273 K)
An-CTF-10-400	425	522	56	406	1.1, 1.84	2.00	3.96
An-CTF-20-400	411	562	69	491	1.02, 1.72	2.13	4.22
An-CTF-10-500	427	573	66	751	1.18, 1.87	2.63	5.22
An-CTF-20-500	449	558	62	700	1.06, 1.66	2.69	5.25

**Table 2:** XPS data of N*_1s_* and O*_1S_* orbitals of synthesized An-CTFs.

Materials	N Species			O Species		
	N-6	N-5	N-Q	C–O	C–OH	H_2_O
An-CTF-10-400	29.51	41.44	29.05	27.89	43.57	28.54
An-CTF-20-400	29.55	40.72	29.73	28.35	42.96	28.69
An-CTF-10-500	29.43	40.62	29.94	28.28	43.44	28.28
An-CTF-20-500	28.94	41.92	29.14	29.35	40.86	29.79

## Data Availability

Not applicable.

## Supplementary Materials

The following supporting information can be downloaded at https://www.mdpi.com/article/10.3390/ijms23063174/s1.
