# Supplementary material for: Ultrastable Covalent Triazine Organic Framework Based on Anthracene Moiety as Platform for High-Performance Carbon Dioxide Adsorption and Supercapacitors
Source: Int J Mol Sci. 2022 Mar 15;23(6):3174. doi: 10.3390/ijms23063174 (PMC8951433; doi:10.3390/ijms23063174)
Supplement: Supplementary file 1 [file ijms-23-03174-s001.zip › ijms-1613837-supplementary.pdf]

Supporting Information

Ultrastable Covalent Triazine Organic Framework Based on Anthracene Moiety as Platforms for High-Performance Carbon Dioxide Adsorption and Supercapacitors

Mohamed Gamal Mohamed[1,2,†], Santosh U Sharma[3,†], Ni-Yun Liu[1],Tharwat Hassan Mansoure[2], Maha Mohamed Samy[1,2], Swetha V Chaganti [3], Yu-Lung Chang [3], Jyh-Tsung Lee[3*], and Shiao-Wei Kuo[1,4,*]

[1]Department of Materials and Optoelectronic Science, Functional Polymers and Supramolecular Materials, National Sun Yat-Sen University, Kaohsiung 80424, Taiwan; mgamal.eldin12@aun.edu.eg (M.G.M.); d083100006@nsysu.edu.tw (M.M.S); m083100007@nsysu.edu.tw (N.Y.L); kuosw@faculty.nsysu.edu.tw (S.-W.K.)
[2]Chemistry Department, Faculty of Science, Assiut University, Assiut 71516, Egypt; mgamal.eldin12@aun.edu.eg (M.G.M.); d083100006@nsysu.edu.tw (M.M.S); tharout.mansour@science.au.edu.eg (T.H.M.)
[3]Department of Chemistry, National Sun Yat-Sen University, Kaohsiung 80424, Taiwan; skshar-ma25086@g-mail.nsysu.edu.tw (S.U.S); d082630006@g-mail.nsysu.edu.tw (S.V.C); yulung@g-mail.nsysu.edu.tw (Y.L.C); jtlee@faculty.nsysu.edu.tw (J. T. L)
[4] Department of Medicinal and Applied Chemistry, Kaohsiung Medical University, Kaohsiung 807, Taiwan; kuosw@faculty.nsysu.edu.tw (S.-W.K.)

Corresponding Author:

Email: jtlee@faculty.nsysu.edu.tw (J. T. Lee) and kuosw@faculty.nsysu.edu.tw (S. W. Kuo)

Authors Contributions

[#]These authors contributed equally to this work.

Material Characterization: the characterization of these as prepared CTFs includes FTIR spectra were collected on a Bruker Tensor 27 FTIR spectrophotometer with a resolution of 4 cm^{-1} by using KBr disk method. ^{13}C nuclear magnetic resonance (NMR) spectra was examined by using an INOVA 500 instrument with DMSO as the solvent and TMS as the external standard. Chemical shifts are reported in parts per million (ppm). The thermal stabilities of the samples were performed by using a TG Q-50 thermogravimetric analyzer under a N_2 atmosphere; the cured sample (ca. 5 mg) was put in a Pt cell with heating rate of 20 °C min^{-1} from 100 to 800 °C under a N_2 flow rate of 60 mL min^{-1}. Wide-angle X-ray diffraction (WAXD) patterns were measured by the wiggler beamline BL17A1 of the National Synchrotron Radiation Research Center (NSRRC), Taiwan. A triangular bent Si (111) single crystal was used to get a monochromated beam having a wavelength (λ) of 1.33 Å. The morphologies of the polymer samples were examined by Field emission scanning electron microscopy (FE-SEM; JEOL JSM7610F) and also by transmission electron microscope (TEM) using JEOL-2100 instrument at an accelerating voltage of 200 kV. BET surface area and porosimetry measurements of samples (ca. 40–100 mg) were measured using BEL Master™/BEL sim™ (v. 3.0.0). N_2 adsorption and desorption isotherms were generated through incremental exposure to ultrahigh-purity N_2 (up to ca. 1 atm) in a liquid N_2 (77 K) bath. Surface parameters were calculated using BET adsorption models in the instrument's software. The pore size of the prepared samples was determined by using nonlocal density functional theory (NLDFT).

Electrochemical Analysis:

Working Electrode Cleaning: Prior to use, the glassy carbon electrode (GCE) was polished several times with 0.05-μm alumina powder, washed with EtOH after each polishing step, cleaned through sonication (5 min) in a water bath, washed with EtOH, and then dried in air.

Electrochemical Characterization: The electrochemical experiments were performed in a three-electrode cell using an Autolab potentiostat (PGSTAT204) and 1 M KOH as the aqueous electrolyte. The GCE was used as the working electrode (diameter: 5.61 mm; 0.2475 cm^2); a Pt wire was used as the counter electrode; Hg/HgO (RE-1B, BAS) was the reference electrode. All reported potentials refer to the Hg/HgO potential. A slurry was prepared by dispersing the sample (45 wt. %), carbon black (45 wt. %), and Nafion (10 wt. %) in a mixture of (EtOH/ H_2O) (200 μL: 800 μL) and then sonicating for 1 h. A portion of this slurry (10 μL) was pipetted onto the tip of the electrode, which was then dried in air for 30 min prior to use. The electrochemical performance was studied through CV at various sweep rates (5–200 mV s^{-1}) and through the GCD method in the potential range from 0 to –1.00 V (vs. Hg/HgO) at various current densities (0.5–20 A g^{-1}) in 1 M KOH as the aqueous electrolyte solution.

The specific capacitance was calculated from the GCD data using the equation

$C_s = (I\Delta t)/(m\Delta V)$ **(S1)**

Where C_s (F g^{-1}) is the specific capacitance of the supercapacitor, I (A) is the discharge current, ΔV (V) is the potential window, Δt (s) is the discharge time, and m (g) is the mass of the NPC on the electrode. The energy density (E, W h kg^{-1}) and power density (P, W kg^{-1}) were calculated using the equations.

$E = 1000C(\Delta V)^2/(2 \times 3600)$ **(S2)**

$P = E/(t/3600)$ **(S3)**

Table S1. Summarized the specific capacitance values of An-CTFs at various scan rate.

Current Density (A g^{-1})	An-CTF-10-400	An-CTF-20-400	An-CTF-10-500	An-CTF-20-500
0.5	435	435	589	552
1	350	331	427	403
2	178	168	382	322
3	123	140	351	270
5	67	139	331	209
10	13	115	296	133
20	2	107	248	79

Table S2. Summarized EIS data fitting of the An-CTF-10-400, An-CTF-20-400, An-CTF-10-500 and An-CTF-20-500.

	Rs (Ω)	Rct (Ω)	CPE-EDL	CPE-P
An-CTF-10-400	9.951	15.37	0.0000410	0.009063
An-CTF-20-400	10.75	28.15	0.0003180	0.006924
An-CTF-10-500	4.068	400	0.0000851	0.755830
An-CTF-20-500	7.08	1110	0.0000214	1.062000

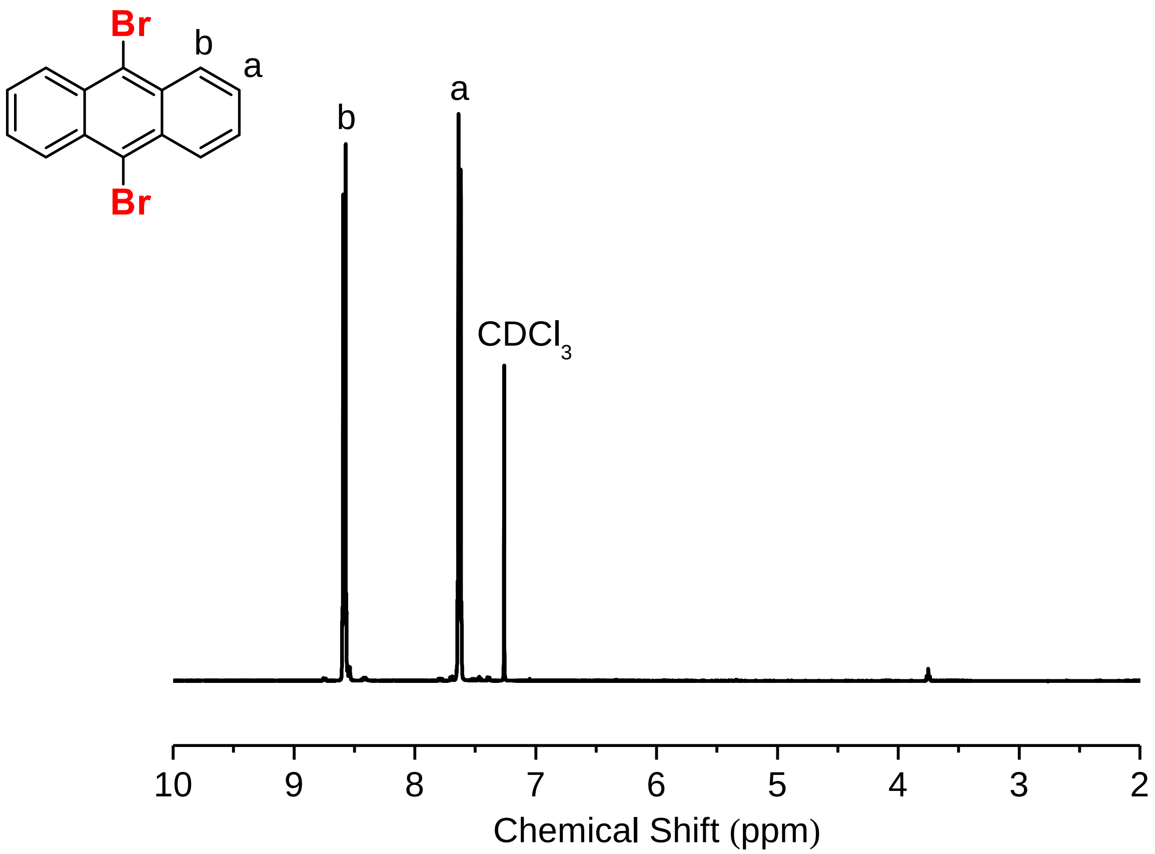

Figure S1. ^{1}H-NMR spectrum of An-Br_2.

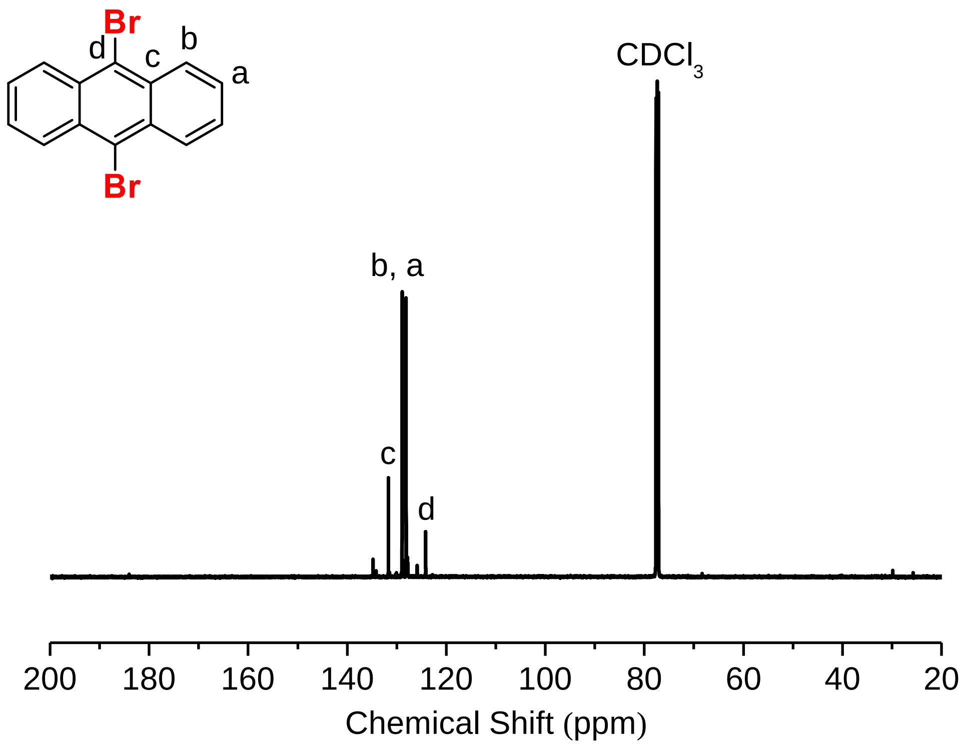

Figure S2. ^{13}C-NMR spectrum of An-Br_2.

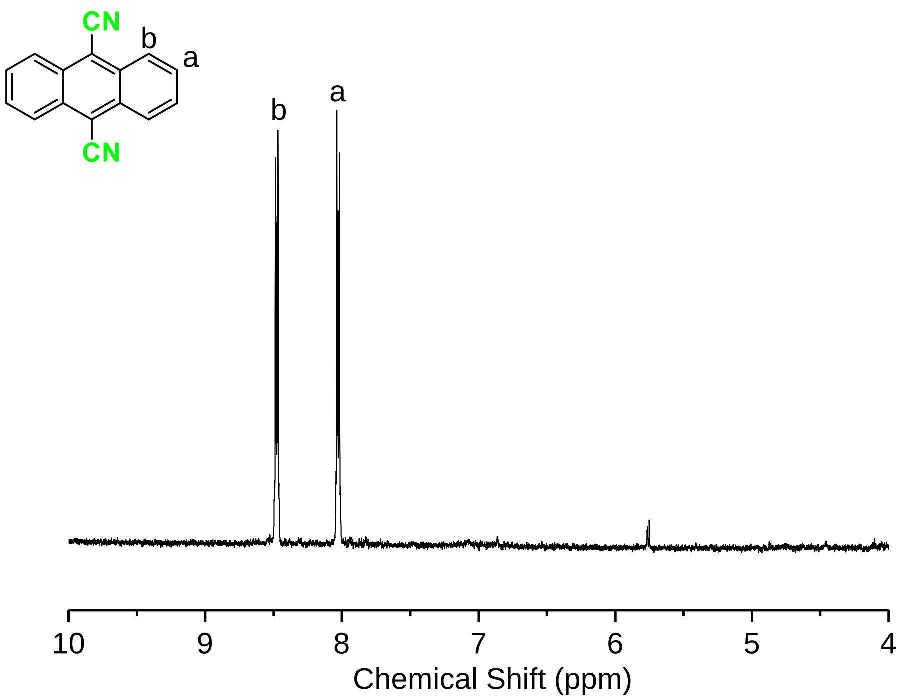

Figure S3. ^{1}H-NMR spectrum of An-CN.

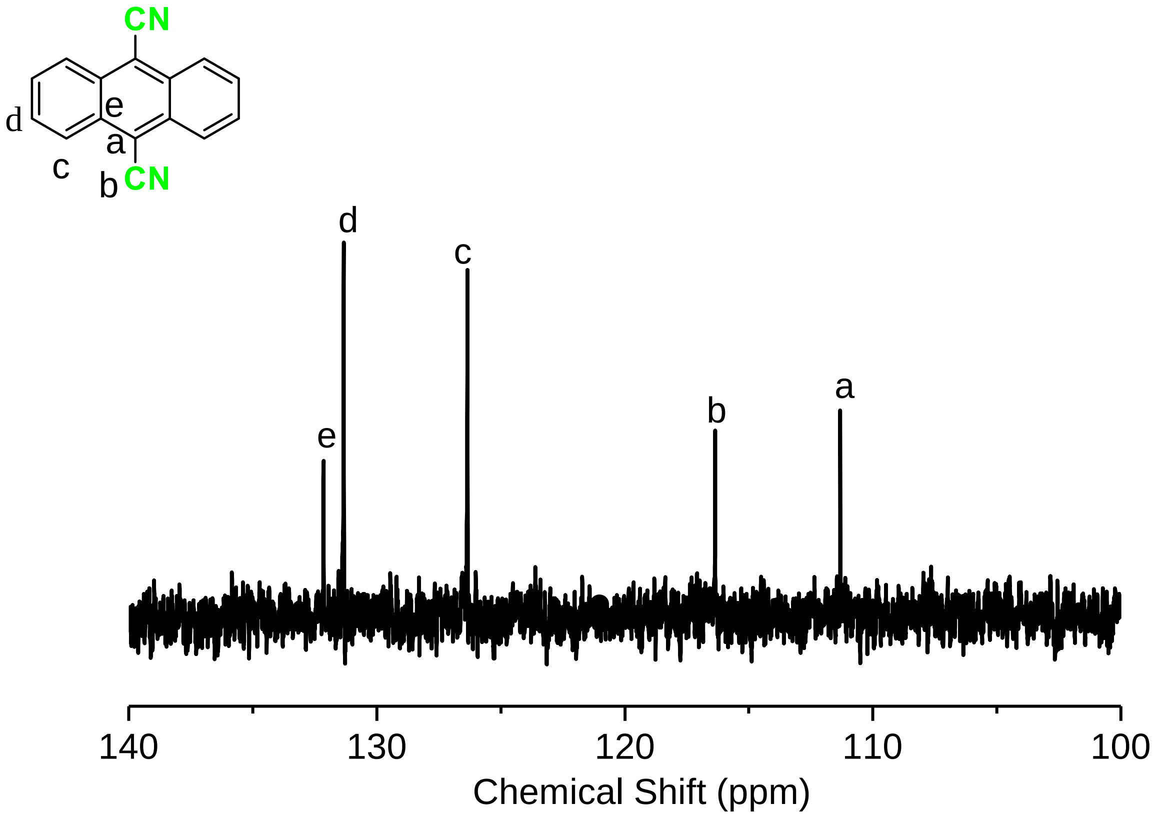

Figure S4. ^{13}C-NMR spectrum of An-CN.

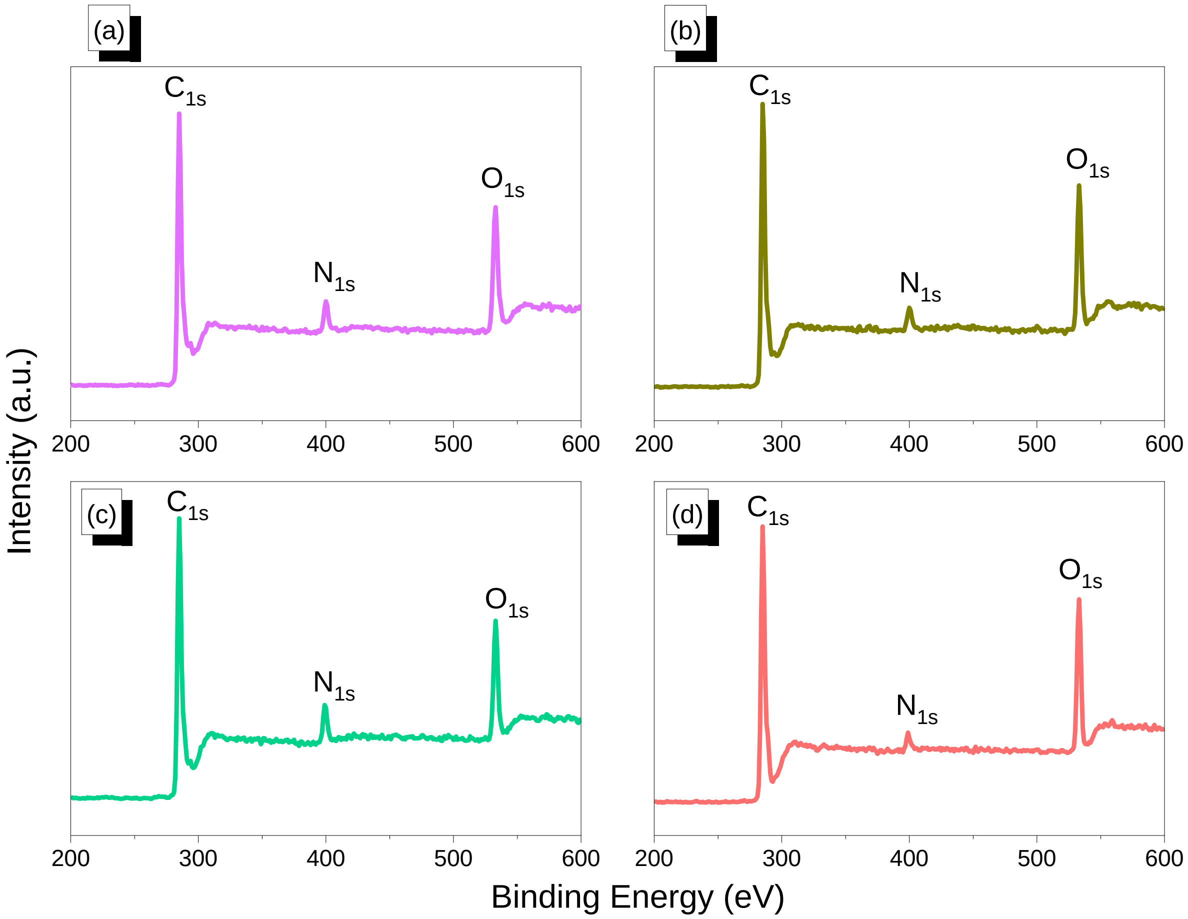

Figure S5. XPS profiles of An-CTF-10-400 (a), An-CTF-20-400 (b), An-CTF-10-500 (c), and An-CTF-20-500 (d).

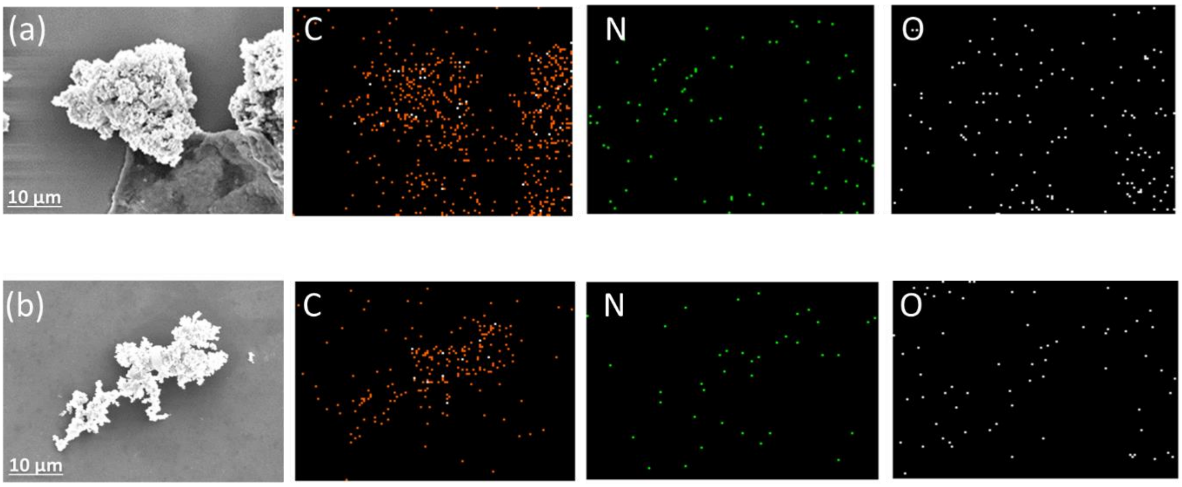

Figure S6. SEM-EDS mapping profiles of An-CTF-10-400 (a), and An-CTF-10-500 (b).

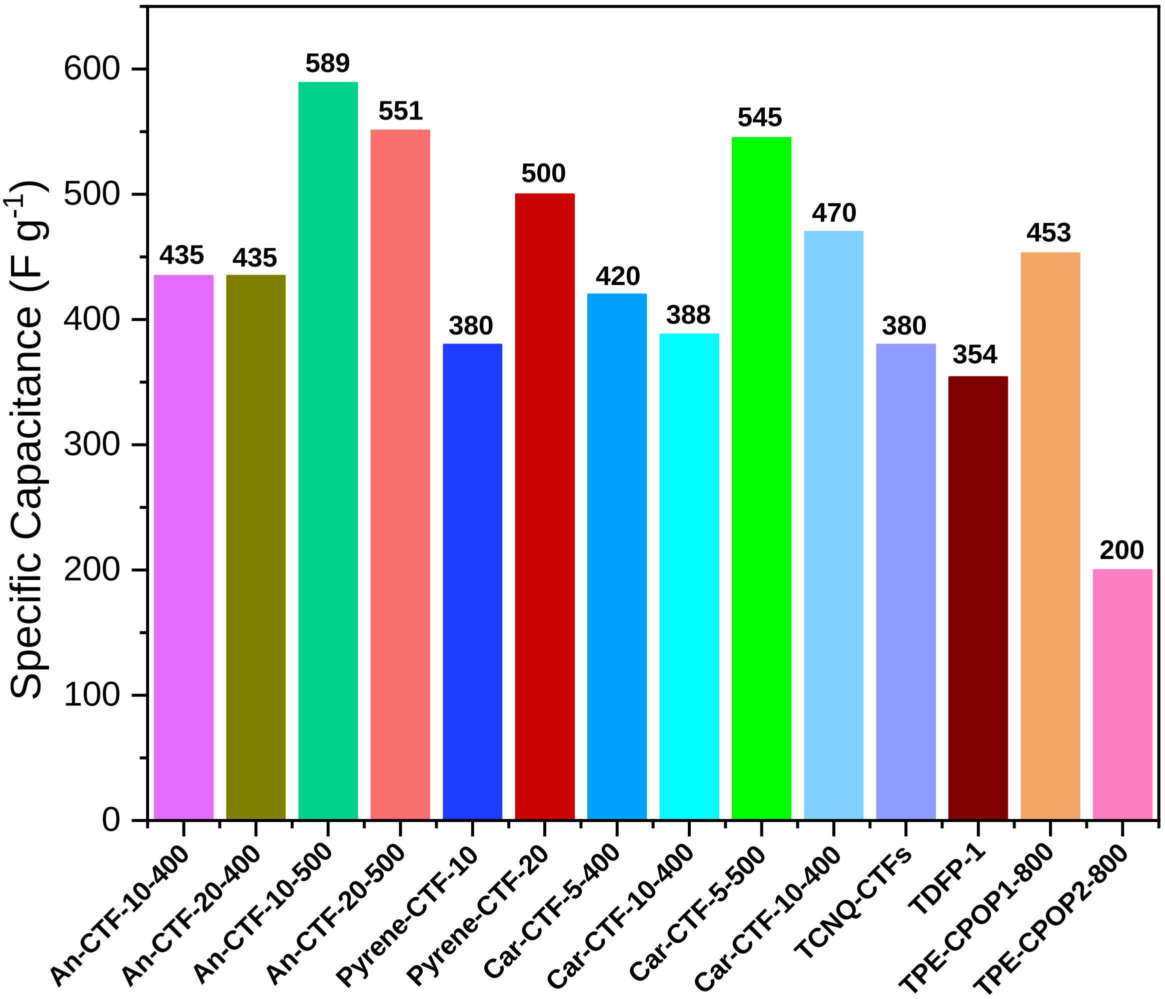

Figure S7. Supercapacitor Performance of An-CTFs materials compared with other reported CTFs materials.
